# Do Non-Glycaemic Markers Add Value to Plasma Glucose and Hemoglobin A1c in Predicting Diabetes? Yuport Health Checkup Center Study

**DOI:** 10.1371/journal.pone.0066899

**Published:** 2013-06-20

**Authors:** Saori Kashima, Kazuo Inoue, Masatoshi Matsumoto, Kimihiko Akimoto

**Affiliations:** 1 Department of Public Health and Health Policy, Institute of Biomedical & Health Sciences, Hiroshima University, Hiroshima, Japan; 2 Department of Community Medicine, Chiba Medical Center, Teikyo University School of Medicine, Chiba, Japan; 3 Department of Community-Based Medical System, Faculty of Medicine, Hiroshima University, Hiroshima, Japan; 4 Akimoto Occupational Health Consultant Office, Tokyo, Japan; University of Verona, Ospedale Civile Maggiore, Italy

## Abstract

**Background:**

Many markers have been indicated as predictors of type 2 diabetes. However, the question of whether or not non-glycaemic (blood) biomarkers and non-blood biomarkers have a predictive additive utility when combined with glycaemic (blood) biomarkers is unknown. The study aim is to assess this additive utility in a large Japanese population.

**Methods:**

We used data from a retrospective cohort study conducted from 1998 to 2002 for the baseline and 2002 to 2006 for follow-up, inclusive of 5,142 men (mean age of 51.9 years) and 4,847 women (54.1 years) at baseline. The cumulative incidence of diabetes [defined either as a fasting plasma glucose (FPG) ≥7.00 mmol/l or as clinically diagnosed diabetes] was measured. In addition to glycaemic biomarkers [FPG and hemoglobin A1c (HbA1c)], we examined the clinical usefulness of adding non-glycaemic biomarkers and non-blood biomarkers, using sensitivity and specificity, and the area under the curve (AUC) of the receiver operating characteristics.

**Results:**

The AUCs to predict diabetes were 0.874 and 0.924 for FPG, 0.793 and 0.822 for HbA1c, in men and women, respectively. Glycaemic biomarkers were the best and second-best for diabetes prediction among the markers. All non-glycaemic markers (except uric acid in men and creatinine in both sexes) predicted diabetes. Among these biomarkers, the highest AUC in the single-marker analysis was 0.656 for alanine aminotransferase (ALT) in men and 0.740 for body mass index in women. The AUC of the combined markers of FPG and HbA1c was 0.895 in men and 0.938 in women, which were marginally increased to 0.904 and 0.940 when adding ALT, respectively.

**Conclusions:**

AUC increments were marginal when adding non-glycaemic biomarkers and non-blood biomarkers to the classic model based on FPG and HbA1c. For the prediction of diabetes, FPG and HbA1c are sufficient and the other markers may not be needed in clinical practice.

## Introduction

For the primary prevention and early intervention of type 2 diabetes, an identification of persons at high risk for developing future diabetes is important. For this purpose, many markers have been identified independently as a predictor or a risk factor and include the classic markers such as blood glucose profiles for the progression to type 2 diabetes. Glycaemic biomarker levels such as plasma glucose at fasting (FPG) [1], [2] and postload [2], [3], late insulin response at postload [4] and hemoglobin A1c (HbA1c) [5]–[7] have been adopted as known biomarkers for predicting type 2 diabetes.

In addition, many other markers (non-glycaemic biomarkers and non-biomarkers) have been proposed as an independent predictor or risk factor for the progression to diabetes in epidemiological studies. First, non-glycaemic biomarkers including in the serum high levels of triglycerides [8], liver enzymes [9]–[15], white blood cell count [16], [17], and C-reactive protein [18]–[20], uric acid [21], [22] and low-density lipoprotein cholesterol [23], [24], high-density lipoprotein cholesterol [25] and creatinine [26] have been reported to predict the risk of development of type 2 diabetes. These epidemiologic studies have shown positive associations between elevated or decreased levels of these risk factors and incident diabetes, independent of classic risk factors such as age, obesity, and fasting and postload plasma glucose levels. Second, non-blood biomarkers or non-biomarkers such as body mass index (BMI), waist-to-hip and waist-to-height ratio, and waist circumference [2], [3], [27], [28], physical inactivity [8], [29], high blood pressure [8], smoking [30] and age [31] have also been reported to be a predictor or a risk factor for diabetes.

These findings of non-glycaemic biomarkers and non-blood biomarkers may reveal the pathogenesis linking these markers with diabetes. However, the question of the clinical utility of these markers as a predictor remains to be solved. The strength of associations expressed in epidemiological terms does not necessarily indicate the clinical usefulness of these markers. What matters most is whether these markers improve the precision of prediction made by glycaemic markers. In other words, biomarker research should be discussed in terms of whether the promise of biomarker research will improve the care of diabetes patients in actual practice [32].

Accordingly, using a large retrospective cohort data set of the Japanese population, we examined whether non-glycaemic biomarkers and non-blood biomarkers improve the predictive power of glycaemic markers to identify future patients of type 2 diabetes using sensitivity and specificity analysis and receiver-operator-characteristic (ROC) curves.

## Materials and Methods

### Study Subjects

**Figure 1:**
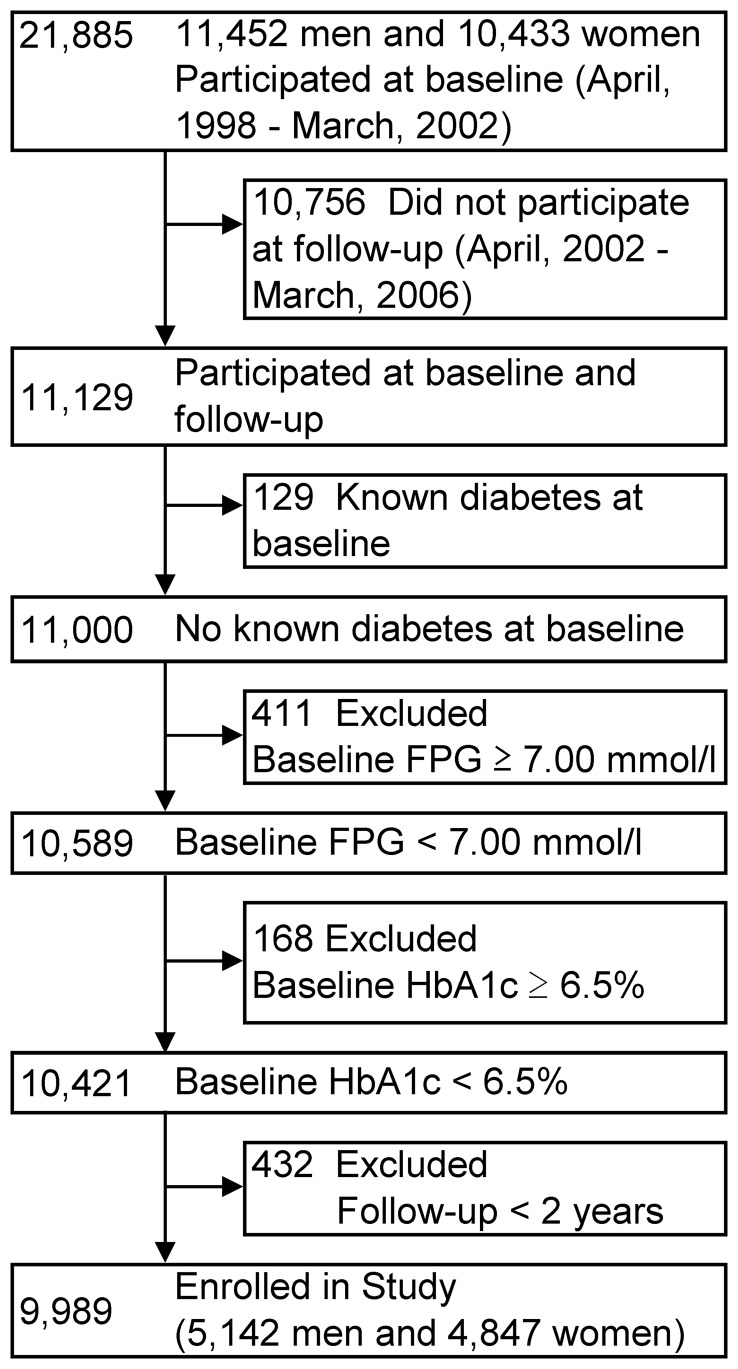
Enrollment of the study subjects. FPG, fasting plasma glucose; HbA1c, hemoglobin A1c.

We used a data set from the health-screening program provided at Yuport Medical Checkup Center in Tokyo during April 1998 and March 2006. The details of this Center’s study have been described elsewhere [6], [33], [34]. During this period, 34,303 persons voluntarily underwent a total of 97,365 checkups. In this study, we set a 4-year baseline period as between April 1998 and March 2002, and the 4-year follow-up period as between April 2002 and March 2006. At the baseline period, 21,885 persons underwent checkups at least once in the total of 47,795 checkups (Figure 1). For repeat participants at the four-year baseline period, the first checkup data was used as the baseline data. During the follow-up period, 23,547 persons underwent a checkup at least once in the total of 49,390 checkups. Total follow-up data was gathered for each person to evaluate incident diabetes.

Next, follow-up data were merged with baseline data, yielding 11,129 persons who had been examined during both time periods. Among them, 129 persons with known diabetes at baseline were excluded and this left a remainder of 11,000 persons. Next, 411 who had a baseline FPG ≥7.00 mmol/l, and in sequence, 168 who had a baseline HbA1c ≥6.5% (National Glycohemoglobin Standardization Program unit) were excluded. Among the remaining 10,421 persons, 432 persons with less than 2 years of follow-up duration between baseline and follow-up checkup were excluded, and finally, 9,989 persons (5,142 men and 4,847 women) were enrolled in this study. All the evaluation procedures were performed in the same manner during the study period, including blood and non-blood measurements. A blood sample was obtained after overnight fasting and measured at the Center’s laboratory.

In accordance with the Private Information Protection Law, information that might identify subjects was safeguarded by the Center. This study was approved by the review board of the Yuport Medical Checkup Center and a written informed consent for anonymous participation in epidemiological research was obtained at every evaluation.

### Diagnosis of Type 2 Diabetes

In all follow-up analyses, type 2 diabetes was defined by a glycaemic biomarker as an FPG level ≥7.00 mmol/l, in accordance with the American Diabetes Association and the Japan Diabetes Society criteria [35], [36], or as a diagnosis of diabetes by a physician sometime between the baseline and follow-up examination.

### Three Types of Markers for Diabetes Prediction

Among all the study subjects, we first divided the markers for progression of diabetes prediction into two groups: blood biomarkers and non-blood biomarkers. Age, BMI, and blood pressure are non-blood biomarkers, and the others are blood biomarkers. The blood biomarkers were then reclassified into two subgroups: glycaemic (blood) biomarkers, non-glycaemic (blood) biomarkers. The classifications of each marker are shown in Table 1.

**Table 1:** Classification of the examined markers to predict diabetes in this study.

Glycaemic (blood) biomarker	Non-glycaemic (blood) biomarker	Non-blood biomarker
Fasting plasma glucose^a^ [1], [2]	Triglycerides [8]	Sex^b^
Hemoglobin A1c^a^ [5]–[7]	Low-density lipoprotein cholesterol [23], [24]	Age [31]
	High-density lipoprotein cholesterol [25]	Body mass index [2], [3], [27], [28]
	Asparate aminotransferase [12], [15]	Systolic blood pressure [8]
	Alanine aminotransferase [9]–[15]	Diastolic blood pressure [8]
	Gamma-glutamyltranspeptidase [9], [11]–[15]	
	White blood cell count [16], [17]	
	Uric acid [21], [22]	
	Creatinine [26]	

a The combination of fasting plasma glucose and hemoglobin A1c were used in the base predictive model for diabetes.

b For the obvious sex difference in the prevalence of diabetes, men and women were separately analyzed.

Each number in a square bracket represents the correspondence to the reference number.

#### Glycaemic biomarkers

For the measurements of FPG and HbA1c levels as glycaemic biomarkers, a Toshiba TBA-40FR Autoanalyzer (Toshiba Medical Systems, Tokyo, Japan) was used. Plasma glucose level was measured via the hexokinase-G6PD method (Denka Seiken, Niigata, Japan) with an inter-assay coefficient of covariation of 3.0% or less. HbA1c level was measured by the latex immuno-agglutinin method (Determiner HbA1c, Kyowa Medex, Tokyo, Japan), with an inter-assay coefficient of covariation of 1.7–2.1%, which was comparable to that of plasma glucose and aligned to the Japan Diabetes Society assigned values. The Japan Diabetes Society value of HbA1c was converted into National Glycohemoglobin Standardization Program units in this study by adding 0.4% [36].

#### Non-glycaemic biomarkers

Non-glycaemic biomarkers including serum levels of lipids and hepatic enzymes, and white blood cell count, uric acid and creatinine level were used to compare with the glycaemic biomarkers (FPG and HbA1c) for the prediction of diabetes. Triglycerides, and total cholesterol and high-density lipoprotein cholesterol were measured using enzymatic methods (reagents supplied by Daiichi Pure Chemicals, Tokyo, Japan). Low-density lipoprotein cholesterol was calculated by Friedewald’s equation [37]. Aspartate aminotransferase (AST) and alanine aminotransferase (ALT) were measured using enzymatic methods (reagents supplied by Denka Seiken, Niigata, Japan), as were gamma-glutamyltranspeptidase (GGT) levels (Wako Junyaku, Osaka, Japan). White blood cell count was measured using the differential count detection method (reagents supplied by Sysmex, Kobe, Japan). Uric acid and creatinine level were measured using enzymatic methods (reagents supplied by Mitsubishi Kagaku Iatron, Tokyo, Japan). All of these markers have been reported to be independent risk factors for diabetes, as previously mentioned [8]–[26].

#### Non-blood biomarkers

The following four non-blood biomarkers were examined for their ability to predict diabetes; age, BMI, and systolic and diastolic blood pressure. BMI was defined as weight divided by height squared (kg/m^2^). Blood pressure was measured by trained nurses using a sphygmomanometer.

### Statistical Analysis

The power of each marker to predict a progression to diabetes was evaluated with a ROC curve. The area under the curve (AUC) of the ROC and a 95% confidence interval (CI) were calculated by the Delong method to evaluate the simple and combined diagnostic utilities of the marker for diabetes prediction [38]. The optimal cut-off point of each marker was determined by calculating the Youden index that maximizes a combination of sensitivity and specificity [39]. Then, its sensitivity, specificity, and positive likelihood ratio for the progression to diabetes were calculated using the cut-off. The likelihood ratio describes how the probability of disease shifts when the finding is present. The higher likelihood ratio means a better test for diagnosis, e.g., a likelihood ratio of “2” means that someone’s positive result would be about 2 times as likely to be seen in someone with a disease than in someone without a disease. In this evaluation, we created following four models: (1) a single marker model, (2) a base model (FPG+HbA1c), (3) an additional model (FPG+HbA1c+non-glycaemic biomarker or non-blood biomarker), and (4) a full model. In the first model (single marker model), we evaluated the predictive ability of the each single marker separately. In the second model (base model), because glycaemic biomarkers (FPG and HbA1c) have been adopted as a component of the criteria for diagnosing diabetes in the guidelines of the American Diabetes Association and the Japan Diabetes Society, a logistic regression equation with those biomarkers as explanatory variables for predicting diabetes was created. The combination of FPG and HbA1c was also reported as a better biomarker for the progression of diabetes than the single FPG biomarker in line the Yuport study [6]. In the third model (additional model), we then added each non-glycaemic biomarker or non-blood biomarker to the base model, and evaluated the predictive ability. In the last model (full model), we entered all the markers (including FPG and HbA1c) into the base model.

The correlations between AST and ALT, and systolic and diastolic blood pressure were high (at 0.82 and 0.90 among men, and 0.82 and 0.89 among women, respectively) which indicates multi-collinearity. Thus, we excluded AST and diastolic blood pressure from the full-model analysis, since the predictive ability of ALT and systolic blood pressure for diabetes was superior to that of AST and diastolic blood pressure at our prior examination, respectively. For each curve, a test for the equality of the AUC of ROC between the base model and the additional model was evaluated using an algorithm suggested by DeLong and Clarke-Pearson [38]. In addition, we calculated a percentage of incremental AUC above 0.5 over the base model (FPG+HbA1c) as 0.5 is an AUC value of the ROC for a diagnostic test which is not better than ‘flipping a coin’ (chance alone).

We conducted separate analysis for men and women because of the gender-difference in anthropometric characteristics and the prevalence of diabetes. All test characteristics and the AUC of ROC was calculated using STATA software (version 12, College Station, TX, USA). Since multiple measures to predict the progression to diabetes are being tested, a statistical *P* value of 0.01 was used to determine statistical significance to reduce the possibility of statistical type I error.

## Results

Over the entire 28,757 person-years of follow-up for men and 26,686 person-years for women, 257 men (5.0%) and 88 women (1.8%) were newly diagnosed as having diabetes. A mean follow-up period was 5.6 (standard deviation: 1.4) years in men with mean age of 51.9 years at baseline and 5.5 (standard deviation: 1.5) in women with mean age of 54.1 years. The baseline characteristics of the men and women study subjects are shown in Table 2. Among them, 226 of men and 75 of women were discovered to have a FPG level ≥7.00 mmol/l and, 9 and 4 were diagnosed as new-onset diabetes by a physician, and 22 and 9 had both, respectively.

**Table 2:** Baseline characteristics of the 9,989 study subjects.

Characteristic	Men (*N* = 5,142)	Women (*N* = 4,847)
Fasting plasma glucose (mmol/l)	5.41±0.49	5.12±0.46
Hemoglobin A1c [mmol/mol (%)]	36±4 (5.4±0.4)	36±4(5.4±0.4)
Age (years)	51.9±11.9	54.1±11.1
Systolic blood pressure (mmHg)	126.0±17.2	121.1±17.9
Diastolic blood pressure (mmHg)	76.8±10.7	72.5±10.8
Body mass index (kg/m^2^)	23.5±2.8	22.3±3.0
Triglycerides (mmol/l)	1.26 (0.90, 1.82)	0.93 (0.69, 1.28)
LDL cholesterol (mmol/l)	3.43±0.83	3.50±0.88
HDL cholesterol (mmol/l)	1.38±0.35	1.67±0.38
Asparate aminotransferase (U/l)	22 (18, 26)	20 (17, 23)
Alanine aminotransferase (U/l)	22 (16, 30)	16 (12, 20)
Gamma-glutamyltranspeptidase(U/l)	23 (15, 41)	11(8, 17)
White blood cell count (10^9^/l)	5.8 (5.0, 6.9)	5.2 (4.5, 6.2)
Uric acid (µmol/l )	364.5±76.3	269.9±60.0
Creatinine (µmol/l )	72.8±11.6	54.1±9.8

Data are expressed as mean ± standard deviation, median (25 percentile, 75 percentile) or number (%).

HDL, high-density lipoprotein; LDL, low-density lipoprotein.

Table 3 shows the predictive ability of the marker in the single marker model among men and women. The AUCs to predict the progression of diabetes among glycaemic biomarkers were 0.874 (95% CI: 0.852–0.896) and 0.924 (95% CI: 0.896–0.952) for FPG, 0.793 (95% CI: 0.767–0.818) and 0.822 (95% CI: 0.777–0.867) for HbA1c, in men and women, respectively. Clearly, glycaemic biomarkers were the best and second-best markers for diabetes prediction, running ahead of the other markers. Among the non-glycaemic biomarkers and non-blood biomarkers, the highest AUC among men was observed at ALT as 0.656 (95% CI: 0.621–0.691), and among women it was at BMI as 0.740 (95% CI: 0.694–0.785). The lowest AUC in both of men and women was creatinine as 0.506 (95% CI: 0.470–0.541) and 0.553 (95% CI: 0.496–0.610). In contrast, all the AUC of non-glycaemic biomarkers and non-blood biomarkers, except for uric acid in men and creatinine in both sexes, were significantly larger than 0.5 (i.e., the 95% CI did not include 0.5), and these markers therefore predicted the progression to diabetes.

**Table 3:** Area under the receiver operating characteristics and predictabilities of single markers for progression of diabetes.^a^

Single marker	AUC (95% CI)	Optimal cutoff point^b^	*N* of test positive (%)	Sensitivity (%)(95% CI)	Specificity (%)(95% CI)	Positive likelihood ratio
Men (*N* = 5,142)						
Fasting plasma glucose	0.874 (0.852–0.896)	≥5.7	1340 (26)	83.7 (78.6–88.0)	77.0 (75.8–78.1)	3.63
Hemoglobin A1c	0.793 (0.767–0.818)	≥37 (5.5)	2050 (40)	80.9 (75.6–85.5)	62.3 (60.9–63.7)	2.15
Triglycerides	0.609 (0.574–0.643)	≥1.55	1818 (35)	52.1 (45.8–58.4)	65.5 (64.2–66.9)	1.51
LDL cholesterol	0.567 (0.532–0.603)	≥3.22	2991 (58)	69.3 (63.2–74.8)	42.4 (41.0–43.8)	1.20
HDL cholesterol	0.577 (0.543–0.611)	≤1.35	2588 (50)	64.6 (58.4–70.4)	50.4 (49.0–51.8)	1.30
Asparate aminotransferase	0.612 (0.576–0.649)	≥22	2657 (52)	68.5 (62.4–74.1)	49.2 (47.8–50.6)	1.35
Alanine aminotransferase	0.656 (0.621–0.691)	≥26	1856 (36)	58.4 (52.1–64.5)	65.1 (63.7–66.4)	1.67
Gamma-glutamyltranspeptidase	0.626 (0.593–0.660)	≥23	2636 (51)	68.5 (62.4–74.1)	49.6 (48.2–51.1)	1.36
White blood cell count	0.573 (0.537–0.609)	≥5.8	2702 (53)	65.0 (58.8–70.8)	48.1 (46.7–49.5)	1.25
Uric acid	0.535 (0.497–0.573)	≥410.4	1404 (27)	35.8 (29.9–42.0)	73.1 (71.9–74.4)	1.33
Creatinine	0.506 (0.470–0.541)	≤88.4	4911 (96)	92.6 (88.7–95.5)	4.3 (3.8–4.9)	0.97
Age	0.547 (0.516–0.578)	≥42	3988 (78)	89.5 (85.1–93.0)	23.1 (21.9–24.3)	1.16
Body mass index	0.640 (0.605–0.676)	≥24.9	1505 (29)	49.0 (42.8–55.3)	71.8 (70.5–73.0)	1.74
Systolic blood pressure	0.569 (0.532–0.606)	≥126	2482 (48)	59.1 (52.9–65.2)	52.3 (50.9–53.7)	1.24
Diastolic blood pressure	0.556 (0.519–0.593)	≥80	2029 (39)	49.0 (42.8–55.3)	61.0 (59.7–62.4)	1.26
Women (*N* = 4,847)						
Fasting plasma glucose	0.924 (0.896–0.952)	≥5.7	490 (10)	80.7 (70.9–88.3)	91.2 (90.4–92.0)	9.16
Hemoglobin A1c	0.822 (0.777–0.867)	≥40 (5.8)	925 (19)	67.0 (56.2–76.7)	81.8 (80.7–82.9)	3.68
Triglycerides	0.684 (0.628–0.739)	≥1.22	1365 (28)	58.0 (47.0–68.4)	72.4 (71.1–73.7)	2.10
LDL cholesterol	0.594 (0.534–0.654)	≥3.67	1972 (41)	54.5 (43.6–65.2)	59.6 (58.2–61.0)	1.35
HDL cholesterol	0.611 (0.555–0.666)	≤1.81	3177 (66)	81.8 (72.2–89.2)	34.8 (33.4–36.1)	1.25
Asparate aminotransferase	0.629 (0.569–0.690)	≥19	2939 (61)	78.4 (68.4–86.5)	39.7 (38.3–41.1)	1.30
Alanine aminotransferase	0.727 (0.675–0.779)	≥17	2161 (45)	80.7 (70.9–88.3)	56.1 (54.7–57.5)	1.84
Gamma-glutamyltranspeptidase	0.648 (0.598–0.698)	≥12	2332 (48)	71.6 (61.0–80.7)	52.3 (50.9–53.7)	1.50
White blood cell count	0.617 (0.560–0.674)	≥5.3	2394 (49)	71.6 (61.0–80.7)	51.0 (49.6–52.4)	1.46
Uric acid	0.622 (0.559–0.685)	≥285.5	1857 (38)	59.1 (48.1–69.5)	62.1 (60.7–63.5)	1.56
Creatinine	0.553 (0.496–0.610)	≤44.2	1239 (26)	35.2 (25.3–46.1)	74.6 (73.4–75.8)	1.39
Age	0.630 (0.581–0.679)	≥51	3160 (65)	86.4 (77.4–92.8)	35.2 (33.8–36.6)	1.33
Body mass index	0.740 (0.694–0.785)	≥22.9	1828 (38)	79.5 (69.6–87.4)	63.1 (61.7–64.4)	2.15
Systolic blood pressure	0.684 (0.635–0.733)	≥124	2068 (43)	71.6 (61.0–80.7)	57.9 (56.5–59.3)	1.70
Diastolic blood pressure	0.642 (0.593–0.691)	≥71	2669 (55)	79.5 (69.6–87.4)	45.4 (44.0–46.8)	1.46

AUC, area under the receiver operating characteristic curve; CI, confidence interval; HDL, high-density lipoprotein; LDL, low-density lipoprotein.

a Diabetes was defined as FPG ≥7.00 mmol/L or known diabetes at follow-up.

b The units of each optimal cutoff point was shown in Table2, respectively.

Table 4 shows the predictabilities of the base model (FPG+HbA1c), and the additional model (FPG+HbA1c+non-glycaemic biomarker or non-blood biomarker) among men and women. The AUC of the base model was 0.895 (95% CI: 0.877–0.914) and 0.938 (95% CI: 0.916–0.960). The incremental AUC above 0.5 over the base model was slightly, but significantly increased by 2.3% from the AUC of the base model in the additional model of ALT (*P* = 0.02), which was the best marker for diabetes prediction in the single model with men excluding the model of glycaemic biomarkers, and 0.2% in the model with BMI (not significant), which was the best such marker in women. The highest AUCs among the additional models with non-glycaemic biomarkers and non-blood biomarkers were, however, observed in the model with ALT in both sexes. Although the differences between the base model and the additional models with HDL-cholesterol and GGT in men were statistically significant, the differences were marginal (incremental AUCs above 0.5 over the base model were 0.4% and −4.0%, respectively). None of the incremental AUCs above 0.5 over the base model showed significant differences in women. Comparing the all-additional models with the base model, the increment in the ROC curves were marginal in both sexes, irrespective of the non-glycaemic biomarkers and non-blood biomarkers enrolled into each model. Regarding the results of the full model, the increments of AUCs above 0.5 from the base model were also marginal (3.3% among men and 0.8% among women, respectively). In this full model, only the three coefficients of FPG, HbA1c and ALT were observed to be significant in predicting diabetes among both sexes.

**Table 4:** The area under the receiver operating characteristics and predictabilities of multiple markers for progression of diabetes^a^ by logistic regression models.^b^

Multiple markers	AUC (95% CI)	*P* value^c^	Incremental AUC above 0.5^d^ (%)	Sensitivity(%) (95% CI)	Specificity(%) (95% CI)	Positive likelihood ratio
**Men (** ***N*** ** = 5142)**						
FPG+HbA1c	0.895 (0.877–0.914)			83.7 (78.6–88.0)	80.5 (79.4–81.6)	4.29
FPG+HbA1c+triglycerides	0.896 (0.878–0.915)	0.42	0.3	84.0 (79.0–88.3)	81.8 (80.6–82.8)	4.61
FPG+HbA1c+LDL-cholesterol	0.895 (0.876–0.914)	0.36	−0.2	84.4 (79.4–88.6)	80.1 (78.9–81.2)	4.23
FPG+HbA1c+HDL-cholesterol	0.897 (0.878–0.915)	<0.01	0.4	84.8 (79.8–89.0)	79.9 (78.7–81.0)	4.22
FPG+HbA1c+ AST	0.898 (0.880–0.916)	0.07	0.7	92.2 (88.2–95.2)	71.9 (70.6–73.2)	3.28
FPG+HbA1c+ALT	0.904 (0.887–0.921)	0.02	2.3	83.7 (78.6–88.0)	83.1 (82.0–84.1)	4.94
FPG+HbA1c+GGT	0.879 (0.858–0.900)	<0.01	−4.0	82.5 (77.3–86.9)	80.2 (79.1–81.3)	4.17
FPG+HbA1c+white blood cell count	0.896 (0.877–0.915)	0.03	0.2	83.3 (78.1–87.6)	81.5 (80.3–82.5)	4.49
FPG+HbA1c+uric acid	0.895 (0.877–0.914)	0.67	0.0	84.4 (79.4–88.6)	80.1 (79.0–81.2)	4.25
FPG+HbA1c+creatinine	0.892 (0.873–0.911)	0.05	−0.9	84.8 (79.8–89.0)	78.9 (77.8–80.1)	4.03
FPG+HbA1c+age	0.896 (0.878–0.915)	0.19	0.3	83.3 (78.1–87.6)	81.5 (80.3–82.5)	4.49
FPG+HbA1c+body mass index	0.898 (0.880–0.916)	0.02	0.8	88.3 (83.8–92.0)	77.4 (76.2–78.5)	3.90
FPG+HbA1c+systolic blood pressure	0.894 (0.876–0.913)	0.07	−0.2	84.8 (79.8–89.0)	79.6 (78.5–80.7)	4.16
FPG+HbA1c+diastolic blood pressure	0.895 (0.876–0.914)	0.95	0.0	83.7 (78.6–88.0)	80.3 (79.2–81.4)	4.25
**Women (** ***N*** ** = 4847)**						
FPG+HbA1c	0.938 (0.916–0.960)			92.0 (84.3–96.7)	81.2 (80.1–82.3)	4.91
FPG+HbA1c+triglycerides	0.940 (0.919–0.961)	0.22	0.4	90.9 (82.9–96.0)	83.6 (82.5–84.7)	5.55
FPG+HbA1c+LDL-cholesterol	0.938 (0.917–0.960)	0.45	0.1	86.4 (77.4–92.8)	87.5 (86.5–88.4)	6.91
FPG+HbA1c+HDL-cholesterol	0.938 (0.917–0.960)	0.61	0.1	89.8 (81.5–95.2)	83.7 (82.6–84.7)	5.49
FPG+HbA1c+AST	0.938 (0.916–0.961)	0.75	0.1	85.2 (76.1–91.9)	88.3 (87.3–89.2)	7.26
FPG+HbA1c+ALT	0.940 (0.919–0.962)	0.11	0.6	93.2 (85.7–97.5)	81.2 (80.1–82.3)	4.96
FPG+HbA1c+GGT	0.938 (0.916–0.960)	0.81	0.0	84.1 (74.8–91.0)	88.9 (88.0–89.8)	7.58
FPG+HbA1c+white blood cell count	0.938 (0.917–0.960)	0.33	0.1	88.6 (80.1–94.4)	84.5 (83.4–85.5)	5.70
FPG+HbA1c+uric acid	0.938 (0.915–0.960)	0.78	0.0	86.4 (77.4–92.8)	87.0 (86.0–87.9)	6.62
FPG+HbA1c+creatinine	0.939 (0.917–0.960)	0.66	0.2	90.9 (82.9–96.0)	83.5 (82.4–84.5)	5.51
FPG+HbA1c+age	0.938 (0.916–0.960)	0.86	0.0	92.0 (84.3–96.7)	80.8 (79.6–81.9)	4.79
FPG+HbA1c+body mass index	0.939 (0.917–0.960)	0.48	0.2	93.2 (85.7–97.5)	80.7 (79.5–81.8)	4.82
FPG+HbA1c+systolic blood pressure	0.938 (0.916–0.960)	0.93	0.0	86.4 (77.4–92.8)	87.2 (86.2–88.1)	6.75
FPG+HbA1c+diastolic blood pressure	0.938 (0.916–0.960)	0.91	−0.4	85.2 (76.1–91.9)	88.5 (87.5–89.4)	7.39

ALT, alanine aminotransferase; AST, asparate aminotransferase; AUC, area under the receiver operating characteristic curve; CI, confidence interval; FPG, fasting plasma glucose; GGT, gamma-glutamyltranspeptidase; HbA1c, hemoglobin A1c; HDL, high-density lipoprotein; LDL, low-density lipoprotein.

a Diabetes was defined as FPG ≥7.00 mmol/L or from known diabetes.

b FPG and HbA1c are placed into all models as the basic predictors.

c *P* value was for comparing the AUC between base model (FPG+HbA1c) and additional models with multiple markers (FPG+HbA1c+non-glycaemic- non-blood biomarker).

d Incremental AUC above 0.5 was incremental AUC above 0.5 over base model (FPG+HbA1c).

**Figure 2:**
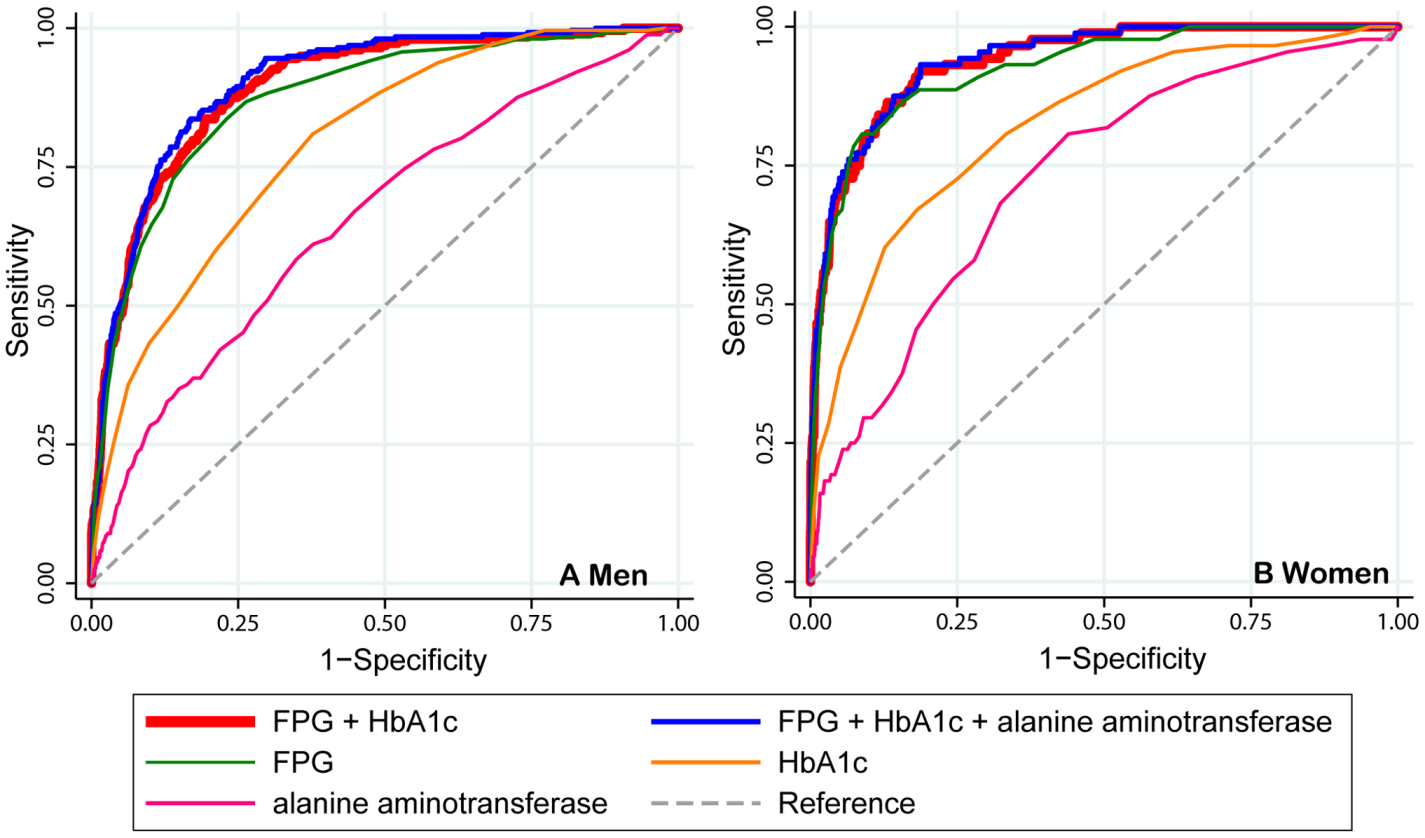
Receiver operating characteristic curves for variables predicting diabetes. The graphs only show glycaemic biomarkers (FPG and HbA1c), and the non-glycaemic biomarker, alanine aminotransferase, which had the highest area under the receiver operating characteristic curves in the additional models. FPG, fasting plasma glucose; HbA1c, hemoglobin A1c.

Figure 2 shows the ROC curve of the single-marker models, the base model (FPG+HbA1c), and the additional model. For simplicity and clarity, the single-marker models and the additional model were shown only with the markers of the glycaemic biomarker (FPG and HbA1c) and ALT, which had the highest AUC in the additional models for each sex. Comparing the additional model with ALT with the base model, the ROC curve was marginal in both sexes.

## Discussion

None of the non-glycaemic biomarkers and non-blood biomarkers showed a substantial improvement in predictive ability for the progression to type 2 diabetes when it was added to the conventional prediction model based on FPG and HbA1c. As indicated in many studies [2], [3], [8]–[28], all of the non-glycaemic biomarkers [serum lipids, liver enzymes, white-blood cell count, and uric acid (only in women)] and non-blood biomarkers (age, blood pressure, and body mass index) examined in this study in some way predicted the progression to diabetes. Although these markers may play some role in the pathogenesis of diabetes, these markers do not appear to add a practical precision to the diagnostic power of plasma glucose and HbA1c levels. The classic glycaemic biomarkers seem to be sufficient as a diagnostic marker in clinical practice.

In this study, additive values of non-glycaemic markers were also evaluated. Many previous studies have evaluated the association between the non-glycaemic markers and future diabetes [2], [3], [8]–[28]. These findings are obviously important in considering the pathogenesis of diabetes. However, whether these markers should be added in practice to classical glycaemic biomarkers in predicting diabetes is, of course, another issue. In our study, for example, ALT independently and substantially predicted a patient’s progression to diabetes, as shown in previous studies [9]–[15]. However, the diagnostic power was not substantially improved when ALT was added to FPG and HbA1c (only a 2.3% AUC increment above 0.5 over the base model for men and 0.63% for women). Sattar et al., have proposed that new biomarker research should focus more on the usefulness of the biomarkers in real clinical practice [32]. Our study is in line with this argument.

In the simple ROC analysis in the single predictor model, the best markers other than glycaemic ones for predicting diabetes were those related with adiposity. The best marker was ALT in men and BMI in women. ALT potentially reflects fatty change in the liver, which is one component of visceral/central adiposity. Visceral/central adiposity is also considered as a risk for diabetes [40], and is more prevalent in men than in women [41]. On the other hand, BMI is an indicator for overall body obesity and might be a better marker for diabetes prediction in women who tend to have subcutaneous (pear-shaped) obesity rather than visceral/central adiposity. It can be noted that non-glycaemic markers have a role in understanding the underlying pathogenesis of diabetes and also in clinical practice even though these markers have little additive value on glycaemic markers for the prediction of diabetes. Although both of the markers did not add substantial value to conventional diagnostic markers, health professionals may be able to refer to these markers in diagnosing or managing diabetes. For example, clinicians may advise high-risk individuals with elevated liver enzymes (such as ALT) or obesity (expressed as a high BMI) to modify lifestyle factors such as diet and exercise and body weight loss.

It is to be noted that the FPG was used both for the prediction and diagnosis of diabetes. In addition, HbA1c is closely related to plasma glucose levels. Because FPG gradually rises from normal to diabetic levels to qualify for the diagnosis of diabetes, individuals already at high FPG levels within the normal range obviously tend to have the highest likelihood of showing a further increase in FPG. Furthermore, these individuals will have the highest likelihood that an increase of a certain magnitude in their FPG eventually will lead to a certain higher level of FPG to match the diagnosis of diabetes compared with individuals having lower values of FPG. Thus, the two glycemic markers (FPG and HbA1c) used in the base model may be referred as self-fulfilling predictors for diabetes. Accordingly, the high odds of glycemic markers (FPG and HbA1c) as predictors for diabetes may be inherent, and it may be expected that the addition of non-glycemic markers will show little or no incremental prediction for diabetes. The advantage of glycemic markers over non-glycemic markers due to a self-fulfilling predictors is a characteristic of this study design.

Several limitations should be mentioned about this study. First, since the study subjects participated on a voluntary basis, they may be healthier than the general population, causing a selection bias. This may have underestimated the incidence of diabetes. However, the 10-year cumulative incidence detected is similar to the estimate derived from a population-based study of middle-aged Japanese [42]. Second, there might be subjects who rapidly progressed to diabetes between the first and second checkups, who therefore were not eligible to participate in this health checkup program thereafter. This would tend to cause an underestimation of the prevalence of diabetes at the second visit. Third, our definition of diabetes favors prediction using FPG over non-FPG and HbA1c, which was indicated in the ROC analysis, i.e., a lesser AUC of HbA1c than of FPG (Table 3 and Figure 2). If the definition using HbA1c was added in the definition of the outcome, more persons would be diagnosed as having diabetes. However, the main research topic is a comparison of the clinical utility of the additional and full models with the base model (FPG+HbA1c). Thus, the conclusion does not appear to be affected substantially by the definition of diabetes. Fourth, similar to the third limitation, at follow-up evaluations, we used a single FPG level for the diagnosis of diabetes. Thus, it is possible that some of the diabetes cases defined in this study had levels higher than the cut off due to chance or the inter-variation of assays. However, it is considered acceptable to be based upon a single fasting glucose measurement for epidemiological estimates of diabetes prevalence and incidence [43] [44]. In addition, data from an oral glucose tolerance test was not obtained in this study, which may cause an underestimation of the true incidence of diabetes. Fifth, not all relevant biomarkers were analyzed, such as vitamin D, adiponectin and other inflammatory markers (eg., high-sensitivity C-reactive protein, interleukins and tumor necrosis factors) other than white blood cell count. For example, higher vitamin D status was associated with decreased risk of type 2 diabetes [45]. However, serum assays of these specialized markers are costly and not common in clinical practice. Furthermore, addition of these inflammatory biomarkers to classic glycaemic biomarkers were in doubt for clinical practice as a resource to predict diabetes [46]. Therefore, the absence of assays of these markers may be justified.

In summary, non-glycaemic biomarkers and non-blood biomarkers gave little or marginal improvement to diagnostic precision when added to the classic predictive model for diabetes using FPG and HbA1c. For the prediction of diabetes, FPG and HbA1c are sufficient, at least, as long as the current diagnostic criteria are used.
